# Chronic glucocorticoid treatment induces hepatic lipid accumulation and hyperinsulinaemia in part through actions on AgRP neurons

**DOI:** 10.1038/s41598-021-93378-3

**Published:** 2021-07-02

**Authors:** Erika Harno, Charlotte Sefton, Jonathan R. Wray, Tiffany-Jayne Allen, Alison Davies, Anthony P. Coll, Anne White

**Affiliations:** 1 Division of Diabetes, Endocrinology and Gastroenterology, Manchester Academic Health Sciences Centre, School of Medical Sciences, Faculty of Biology, Medicine and Health, University of Manchester, 3.020 AV Hill Building, Manchester, M13 9PT UK; 2 MRC Metabolic Diseases Unit, Wellcome-MRC Institute of Metabolic Science, University of Cambridge, Cambridge, CB2 0QQ UK; 3 Division of Diabetes, Endocrinology and Gastroenterology, Manchester Academic Health Sciences Centre, School of Medical Sciences, Faculty of Biology, Medicine and Health, University of Manchester, 3.016 AV Hill Building, Manchester, M13 9PT UK

**Keywords:** Neuroscience, Feeding behaviour, Metabolic diseases, Neuroendocrinology, Endocrine system and metabolic diseases

## Abstract

Glucocorticoids (GCs) are widely prescribed anti-inflammatory medicines, but their use can lead to metabolic side-effects. These may occur through direct actions of GCs on peripheral organs, but could also be mediated by the hypothalamic AgRP neurons, which can increase food intake and modify peripheral metabolism. Therefore, the aim of this study was to examine the metabolic effects of chronic treatment with the GC corticosterone (Cort, 75 μg/ml in drinking water) in mice lacking the glucocorticoid receptor (GR) on AgRP neurons. Female AgRP-GR KO mice had delayed onset of Cort-induced hyperphagia*.* However, AgRP-GR KO had little impact on the increased body weight or adiposity seen with 3 weeks Cort treatment. Cort caused hepatic steatosis in control mice, but in Cort treated female AgRP-GR KO mice there was a 25% reduction in liver lipid content and lower plasma triglycerides. Additionally, Cort treatment led to hyperinsulinaemia, but compared to controls, Cort-treated AgRP-GR KO mice had both lower fasting insulin levels and lower insulin levels during a glucose tolerance test. In conclusion, these data indicate that GCs do act through AgRP neurons to contribute, at least in part, to the adverse metabolic consequences of chronic GC treatment.

## Introduction

Glucocorticoids (GCs) are widely prescribed medicines for the treatment of inflammatory disorders including arthritis, asthma and some malignancies, with 12% of patients in England being prescribed oral GCs in 2018^1^. Chronic treatment of patients with GCs, especially at high doses, is associated with a plethora of side effects. These include body weight gain^2^, central obesity, insulin resistance and an increased risk of developing diabetes mellitus^3^. While some side effects such as osteoporosis can be reduced with the use of prophylactic agents, at present there are no similar treatments for the metabolic effects. Therefore, delineating the mechanisms of the metabolic effects from those of the beneficial anti-inflammatory effects, could lead to the design of alternative GCs or co-therapies to reduce the metabolic side effects.

Model organisms have proven to be useful to further understand the mechanisms that underpin the effect of exogenous GCs on body weight and body composition. Although there are recognised differences in study outcome depending upon rodent species and the type and formulation of GC used^4–7^, data from murine models have highlighted that the increase in food intake and altered adiposity often seen with GC therapy are likely to be mediated via actions both in the brain and at the level of peripheral tissues. For example, GCs are recognised to act in the arcuate nucleus of the hypothalamus to induce hyperphagia^8–11^, leading to increased body weight. Peripherally, GCs act on adipose tissue and can regulate de novo lipogenesis and induce lipolysis depending on the physiological or pharmacological context (reviewed in Macfarlane et al. 2008^12^). This can cause excess adiposity or adipose tissue remodelling and fat redistribution respectively^8–13^. Furthermore, in brown adipose tissue (BAT), GCs are known to cause lipid infiltration, as well as reduce the expression of markers of thermogenesis, such as UCP-1^9,14^. While the effects of chronic GCs acting on BAT have not been fully elucidated, perturbation of heat production in BAT has the potential to reduce energy expenditure and therefore enhance the development of obesity.

Another major metabolic side-effect of GCs is disrupted glucose homeostasis. This has been well characterised in both human populations and model organisms (both mouse and rat) exposed to GCs^4, 15–18^. It appears to be as a result of direct and indirect actions (via regulation of other hormones) at a number of sites. The direct actions of GCs on a range of peripheral tissues such as skeletal muscle, adipose tissue, liver and pancreas are well known to contribute to glucose intolerance and a reduction in insulin sensitivity (reviewed in Pasieka et al. 2016^19^ and Rafacho et al. 2014^20^).

GC action within the brain is also likely to contribute to the adverse effects on glucose and insulin metabolism. In particular, GCs act on AgRP neurons within the hypothalamus, and there is a wide body of evidence demonstrating that AgRP plays a critical role in the control of energy balance^21–25^. In addition, AgRP is also well-recognised to be able to affect metabolic function in peripheral organs (reviewed in Ruud et al. 2017^26^). For example, acute activation of AgRP neurons, using an optogenetic approach, results in insulin resistance^27^, and insulin receptor deletion from these neurons leads to improved glucose homeostasis^28^. Furthermore, deletion of the metabolic flexibility regulating enzyme, carnitine acetyltransferase (Crat), in AgRP neurons increases liver triglycerides^29^, demonstrating that manipulation of AgRP neurons can affect hepatic lipid dynamics.

We have previously reported that GC excess in male mice leads to hyperphagia, obesity and hyperinsulinaemia. This state is characterised by elevated *Agrp* mRNA and AgRP is known to be a potent orexigenic neuropeptide produced in the arcuate nucleus of the hypothalamus. Our previous studies have shown that deletion of AgRP only has a minimal effect on the metabolic phenotype observed when corticosterone (Cort) is given in drinking water to male mice^9^. However, AgRP neurons also express other potent neuropeptides and transmitters, and other reports have clearly shown important roles for these neurons that are not dependent upon drive by AgRP peptides alone^22,30,31^. To further investigate the role of these neurons in the development of GC-induced side effects, we studied the effects of GCs on food intake, adiposity, hepatic lipid balance and glucose homeostasis in male and female mice lacking GR on AgRP neurons. Our data demonstrate that AgRP neurons have a role in mediating the effects of GCs on hepatic steatosis and insulin homeostasis.

## Results

### GR is knocked down in AgRP neurons in AgRP-GR KO mice

**Figure 1:**
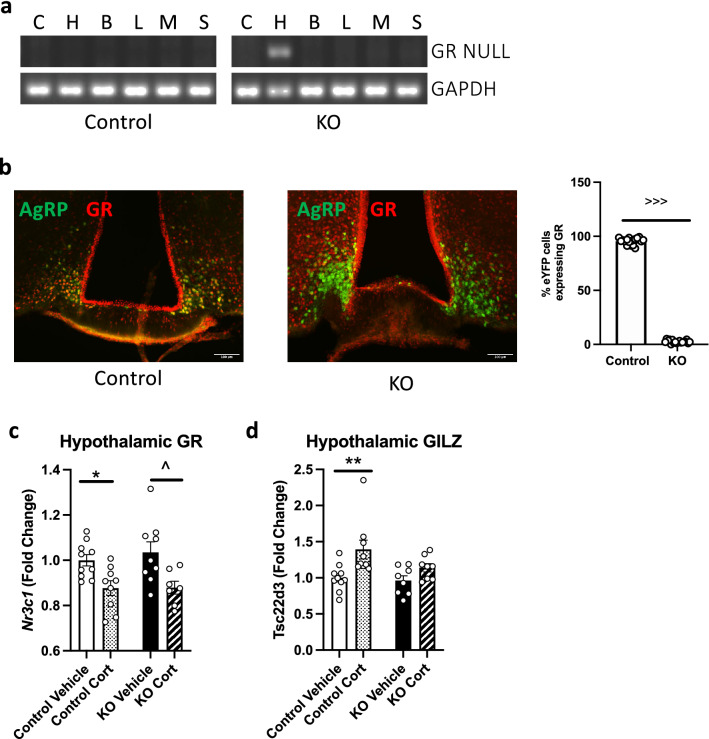
GR is knocked down in AgRP neurons. (**a**) Recombination of the NULL band is only present in the hypothalamus of male AgRP-GR KO mice. (**b**) Representative image showing co-localisation of GR and eYFP (AgRP) in the arcuate nucleus of female control, but not female AgRP-GR KO mice. Graphical insert showing the percentage co-localisation of GR and AgRP. (**c**) *Nr3c1* (GR) and (**d**) *Tsc22d3* (GILZ) mRNA expression in the whole hypothalamus of vehicle and 3 week Cort treated female control and AgRP-GR KO mice. C: cerebellum, H: hypothalamus, B: brown adipose tissue, L: liver, M: skeletal muscle, S: subcutaneous adipose tissue. (**a**) n = 1, (**b**) n = 4, graphical representation of 18 sections per genotype, unpaired student’s *t*-test (**c**) and (**d**) n = 7–10, Mann–Whitney non-parametric t-tests. *P < 0.01, **P < 0.01 control vehicle versus Cort. ^*P* < 0.05 KO vehicle versus KO Cort. ^>>>^*P* < 0.001 control versus KO.

To generate mice with the glucocorticoid receptor (GR; *Nr3c1*) deleted solely from AgRP neurons, we crossed GR^flox/flox^ with AgRP^-IRES-Cre^ mice to obtain AgRP^-IRES-Cre/+^::GR^flox/flox^ mice (hereinafter AgRP-GR KO). To show specificity of the knockdown, we amplified the GR NULL band in a range of tissues including cerebellum, hypothalamus, liver and skeletal muscle and found recombination only in the hypothalamus (Fig. 1a). To further demonstrate the knockdown of GR on AgRP neurons, we carried out dual immunofluorescence to identify co-localisation of AgRP and GR. We further crossed our AgRP-GR KO strain to a Rosa-26-eYFP line to visualise the AgRP neurons, as we have found commercially available AgRP antibodies to be non-specific in the *Agrp*-null mice. In AgRP^CRE/+^::ROSA26^+/?^::GR^+/+^ control mice, dual staining with eYFP marking AgRP neurons showed 95% co-localisation with GR and AgRP, and there was a 97% knockdown of GR in AgRP-GR KO mice (Fig. 1b). When the level of *Nr3c1* mRNA expression in the whole hypothalamus was examined, no difference in expression was observed between control and AgRP-GR KO mice with or without 3 weeks corticosterone (Cort; 75 μg/ml) treatment (Fig. 1c) and both control and AgRP-GR KO mice demonstrated a decrease in GR expression with Cort treatment. Lastly, we investigated GR activation using *Tsc22d3* (glucocorticoid-induced leucine zipper; GILZ) as a marker. Cort treatment increased GILZ expression in the whole hypothalamus of control mice, but not in AgRP-GR KO mice (Fig. 1d).

We next assessed whether the expected peripheral effects of GCs were maintained in peripheral tissues of AgRP-GR KO mice after 3 weeks Cort treatment. GCs are recognised to cause atrophy of various tissues including the adrenal glands, spleen and skeletal muscle. A similar degree of loss of mass was observed in these tissues in both control and AgRP-GR KO mice (Supplemental Figure S1a–S1c). Additionally, *Tsc22d3* mRNA expression was increased as expected in both liver and adipose tissue in control and AgRP-GR KO mice (Supplemental Figure S1d and S1e), demonstrating that GCs are still active in these peripheral tissues. Furthermore, both control and AgRP-GR KO mice treated with Cort had similar circulating corticosterone levels, sampled prior to stress influencing the endogenous corticosterone levels (Supplemental Figure S1f) and similar water intake [indicative of the amount of Cort administered) (Control Cort 129.7 ± 9.6 ml vs. KO Cort 144.3 ± 18.5 ml, *P* = ns)]. These results indicate that both genotypes of mice had similar exposure to GCs.

### Delayed onset hyperphagia in Cort treated female AgRP-GR KO mice

**Figure 2:**
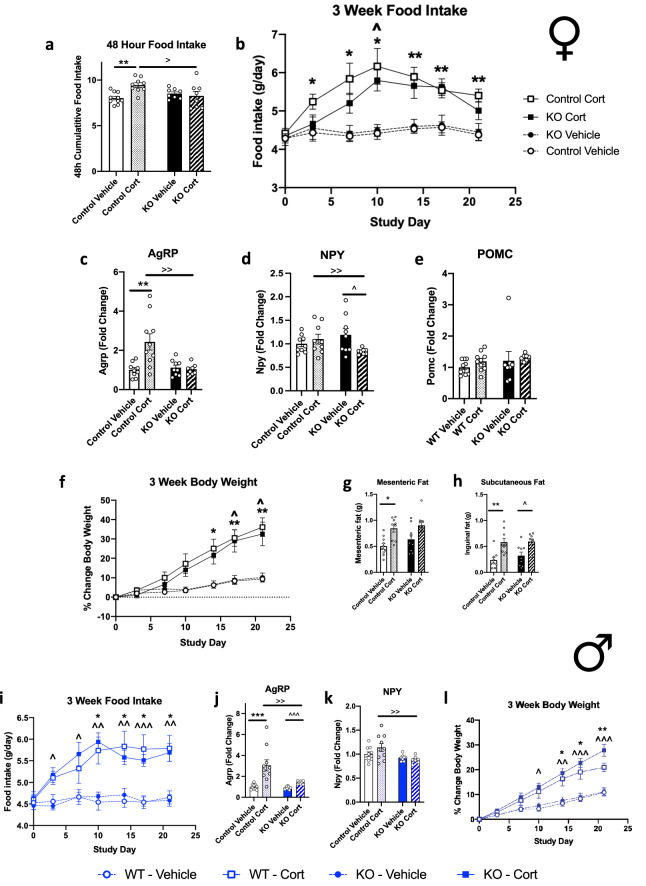
(**a**–**h**) Female AgRP-GR KO mice have delayed onset hyperphagia, but normal body weight gain with corticosterone (Cort) treatment. (**a**) 48 h, (**b**) 3 week food intake. (**c**) *Agrp*, (**d**) *Npy* and (**e**) *Pomc* mRNA expression in whole hypothalamus, (**f**) percent change in body weight, (**g**) mesenteric and (**h**) subcutaneous adipose tissue wet weight in female control and AgRP-GR KO mice treated with Cort in drinking water for 3 weeks. (**i**–**l**) Male mice. (**i**) food intake, (**j**) *Agrp* and (**k**) *Npy* mRNA expression in whole hypothalamus and (**l**) percent change in body weight in male control and AgRP-GR KO mice treated with Cort in drinking water for 3 weeks. (**a**–**h**) n = 7–10, (**i**–**l**) n = 7–10. (**a**, **b**, **f**, **g**, **h**, **i**, **l**) Two-way ANOVA with Tukey Multiple Comparison test, (**c**, **d**, **e**, **j**, **k**) Mann–Whitney non-parametric t-tests. *P < 0.01, **P < 0.01, ***P < 0.001 control vehicle versus Cort, ^*P* < 0.05, ^^*P* < 0.01, ^^^*P* < 0.001 KO vehicle versus Cort. ^>^*P* > 0.05, ^>>^*P* < 0.01 control Cort versus KO Cort.

Previous studies have shown little effect on body weight and appetitive behaviours from loss of GR on AgRP neurons under chow and high fat diet conditions^32^. However, we were interested in the direct effects of GCs acting on these neurons, so we treated male and female mice with Cort in drinking water for three weeks to assess the effect on the metabolic phenotype of excess GCs acting on AgRP neurons. Cort treatment increased food intake in female control mice in the first 48 h, however this increased food intake was absent in AgRP-GR KO mice (Fig. 2a). Over three weeks, the female control mice maintained their Cort-induced hyperphagia (Fig. 2b) as shown previously in male C57Bl/6J mice. AgRP-GR KO mice, however, had delayed onset Cort-induced hyperphagia, but by day 10 were eating a similar amount to Cort-treated control mice (Fig. 2b), suggesting that other neuronal populations are responsible for the sustained hyperphagic effect of Cort treatment.

### Cort-induced increases in Agrp expression are prevented in female AgRP-GR KO mice

Given that in the AgRP-GR KO mice, GCs are not able to act directly on AgRP neurons, we investigated the potential effects on the neuropeptides expressed by this neuronal population after Cort treatment. After 2 days, the levels of *Agrp* in whole hypothalami of AgRP-GR KO mice were significantly less than those in the control mice (Supplemental Figure S2a). Cort treatment for 3 weeks increased *Agrp* mRNA expression in control female mice, but this increase did not occur when GR was deleted from AgRP neurons (Fig. 2c).

Cort treatment did not change the expression of either *Npy* or *Pomc* mRNA in control or AgRP-GR KO mice after 2 days (Supplemental Figure S2b and S2c). In addition, Cort treatment did not increase *Npy* (Fig. 2d) or decrease *Pomc* (Fig. 2e) in these groups at 3 weeks.

Despite the delayed onset of hyperphagia with Cort treatment, body weight increased by a similar trajectory over the three week study in female AgRP-GR KO and control mice (Fig. 2f), accompanied by a similar feed efficiency (Supplemental Figure S2d). Furthermore, terminal adipose tissue mass was similar between Cort treated control and AgRP-GR KO mice (Fig. 2g,h).

### Cort-induced hyperphagia and obesity persists in male AgRP-GR KO mice

Male control (GR^flox/flox^) mice had elevated food intake, body weight and adiposity (Fig. 2i,l and Supplemental Figure S3) in response to Cort, which is a similar metabolic response to C57Bl/6J mice^9^. However, male AgRP-GR KO mice, unlike female AgRP-GR KO mice, had no protection from early Cort-induced hyperphagia (Fig. 2i) and this was despite a much smaller increase in *Agrp* expression with Cort treatment compared to vehicle-treated mice (Fig. 2j). There was also decreased *Npy* expression between Cort-treated control and AgRP-GR KO mice (Fig. 2k). Furthermore, compared to their control counterparts, male AgRP-GR KO mice had similar weight gain (Fig. 2l), and increases in adiposity (Supplemental Figure S3a) and brown adipose tissue (BAT) weight (Supplemental Figure S3b) after 3 weeks Cort treatment. Interestingly, the liver weight of male AgRP-GR KO mice was not increased with Cort treatment (Supplemental Figure S3c).

### Cort treatment modifies brown adipose tissue in both control and AgRP-GR KO female mice

Activation of AgRP neurons can affect BAT^27^ and GCs induce “whitening” of BAT^9, 14^. In the present study, three weeks Cort treatment increased BAT weight, as expected, in control mice, and there was a similar increase in the weight of BAT in AgRP-GR KO mice (Supplemental Figure S4a). We additionally examined whether there were any differences in genes involved in thermogenesis in BAT. Of the four genes we investigated (*Ppargc1a*, *Ucp1*, *Prdm16* and *Cidea*), the change in expression in response to Cort was similar between control and AgRP-GR KO mice (Supplemental Figure S4b). This indicates that the Cort-induced changes in BAT weight are not mediated via AgRP neurons in female mice.

### Female AgRP-GR KO mice have less GC-induced hepatic steatosis

**Figure 3:**
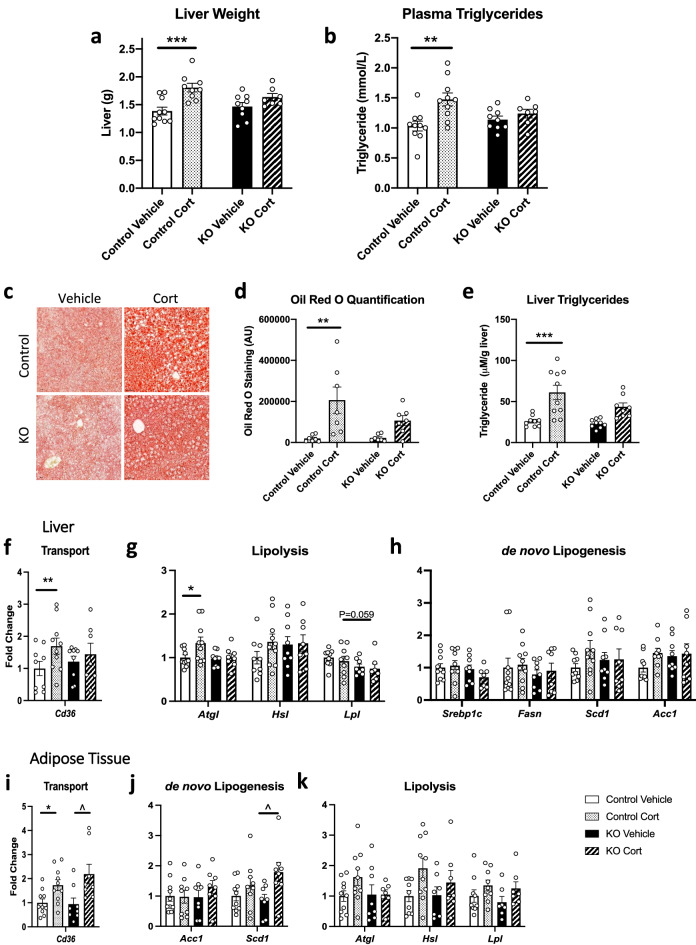
Female AgRP-GR KO mice are partially protected from corticosterone (Cort)-induced hepatic steatosis. (**a**) Liver weight, (**b**) plasma triglycerides, (**c**) representative images of Oil Red O staining, (**d**) quantification of Oil Red O staining, (**e**) liver triglycerides, mRNA expression in liver of (**f**) *Cd36*, a fatty acid transport gene, (**g**) lipolysis genes and (**h**) de novo lipogenesis genes. Adipose mRNA expression of (**i**) *Cd36*, a fatty acid transport gene, (**j**) de novo lipogenesis and (**k**) lipolysis genes. All mice were treated with vehicle or Cort in drinking for 3 weeks. (**a**) and (**b**) n = 7–10, Two-way ANOVA with Tukey Multiple Comparison test, (**d**) n = 7, 30 images per animal analysed, Two-way ANOVA with Tukey Multiple Comparison test. (**e**) n = 8–10, Two-way ANOVA with Tukey Multiple Comparison test, (**f**–**k**) n = 7–10, Mann–Whitney non-parametric t-tests. *P < 0.01, **P < 0.01, ***P < 0.001 control vehicle versus Cort, ^*P* < 0.05 KO vehicle versus Cort.

Liver weight and fat deposition were increased with 3 weeks Cort treatment in female mice (Fig. 3a,c), as was also shown in their male littermates (Supplemental Figure S3c). Furthermore, in female, as in the male, AgRP-GR KO mice, there was no increase in liver weight with Cort treatment (Fig. 3a). Chronic Cort treatment also increased plasma triglycerides in control mice at three weeks (Fig. 3b), but this was absent in AgRP-GR KO mice treated with Cort (Fig. 3b). In control mice, Cort also increased hepatic lipid content, as measured by Oil Red O (Fig. 3c,d) and liver triglycerides (Fig. 3e). This effect was less pronounced in AgRP-GR KO mice (Fig. 3c–e), giving a greater than 25% reduction in hepatic triglycerides compared to Cort-treated control mice (Fig. 3e).

To investigate the underlying mechanisms behind the improved plasma triglycerides and hepatic steatosis we examined the expression of some of the genes involved in lipid homeostasis in liver and subcutaneous (inguinal) adipose tissue. In liver, the expression of the fatty acid transporter, *Cd36* was increased with Cort treatment in control, but not AgRP-GR KO mice (Fig. 3f). The mRNA expression of *Atgl,* a gene involved in lipolysis, was increased in Cort treated control, but not in AgRP-GR KO mice compared to their respective vehicle groups (Fig. 3g). There were no obvious changes in the gene expression of the regulators of de novo lipogenesis, between Cort-treated control and AgRP-GR KO mice (Fig. 3h).

In inguinal adipose tissue, the mRNA expression of *Cd36* was increased similarly in both control and AgRP-GR KO mice with Cort treatment (Fig. 3i), whereas the mRNA expression of *Scd1,* a gene involved in de novo lipogenesis, was increased in Cort-treated AgRP KO mice compared to Cort treated control mice (Fig. 3j). There were no significant increases in the mRNA expression of genes controlling lipolysis in either control or AgRP-GR KO adipose tissue (Fig. 3k).

### AgRP-GR KO mice on Cort are less hyperinsulinaemic than control mice on Cort

**Figure 4:**
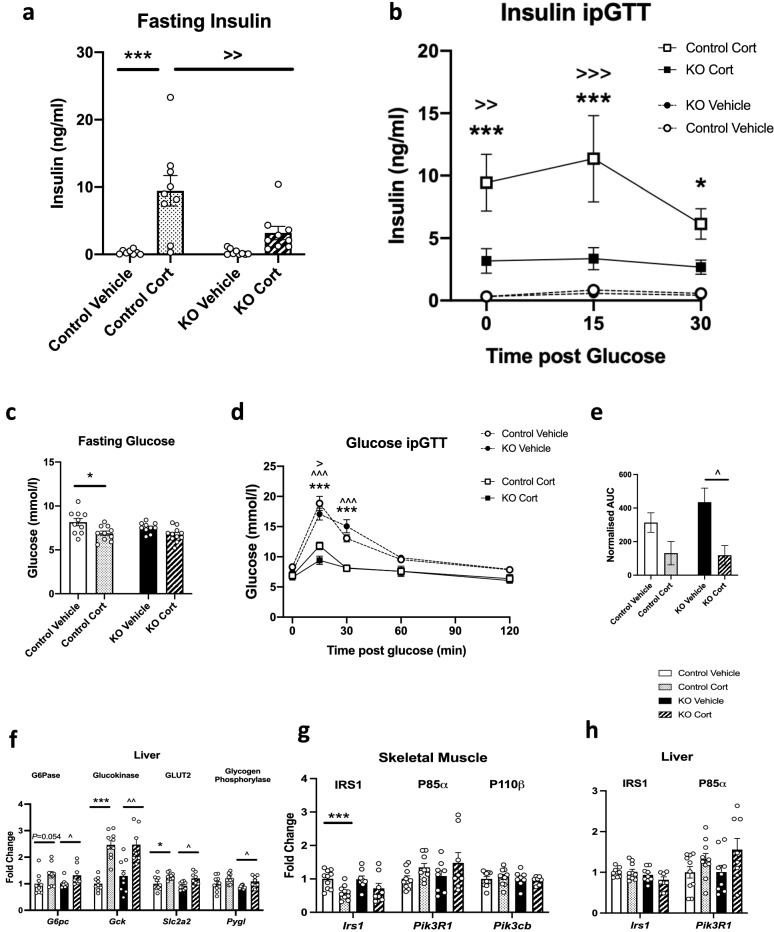
Female AgRP-GR KO mice are partially protected from corticosterone (Cort)-induced insulin resistance. (**a**) Fasting insulin following a 5 h day time fast in control and AgRP-GR KO mice treated with Cort for 10 days. (**b**) Insulin concentrations during an intraperitoneal glucose tolerance test (ipGTT) following 10 days Cort treatment. (**c**) Fasting glucose, (**d**) glucose levels during an intraperitoneal glucose tolerance test (ipGTT), (**e**) area under the curve of the glucose ipGTT undertaken following 10 days Cort treatment, (**f**) mRNA expression of genes associated with gluconeogenesis in liver after 3 weeks Cort treatment. mRNA expression of genes associated with insulin resistance in (**g**) skeletal muscle and (**h**) liver. (**f**–**h**) Samples were taken from non-fasting animals (**a**) n = 8–10, (**b**) n = 8–9, (**c**–**e**) 8–10 Two-way ANOVA with Tukey Multiple Comparison test, (**f**–**h**) n = 7–10, Mann–Whitney non-parametric t-tests. *P < 0.01, ***P < 0.001 control vehicle versus Cort, ^*P* < 0.05, ^^*P* < 0.01 KO, ^^^*P* < 0.001 vehicle versus Cort, ^>^*P* < 0.05, ^>>^*P* > 0.01, ^>>>^*P* > 0.001 control Cort versus KO Cort.

To assess glucose tolerance, an intraperitoneal glucose tolerance test (ipGTT) was carried out in a separate cohort of female mice treated with Cort for 10 days. Ten days of treatment was chosen as at this timepoint there was no difference between Cort-induced food intake (Fig. 2b) and body weight (Fig. 2f) between control and AgRP-GR KO mice. Cort treatment increased fasting insulin levels in both control and KO mice (Fig. 4a), but the effect was much greater in control animals suggesting that loss of GR on AgRP neurons decreases Cort-induced insulin resistance.

During the ipGTT, this marked difference in degrees of Cort-induced hyperinsulinaemia between AgRP-GR KO and control animals was maintained (Fig. 4b). Of note, throughout the GTT there was little change in the insulin levels in Cort treated AgRP-GR KO mice and although the mean levels of insulin in this group were around 5.8 fold higher than vehicle treated AgRP-GR KO animals, the difference did not reach statistical significance.

In terms of glucose there was no difference in fasting glucose before the ipGTT (Fig. 4c). Levels during the GTT, the most striking effect was driven by Cort treatment rather than genotype, with a significantly lower glucose at 15 and 30 min seen in Cort treated animals of both genotypes compared to vehicle (Fig. 4d) leading to a reduction in AUC, which was more marked in AgRP-GR KO mice (Fig. 4e). Of note, glucose levels at 15 min in Cort treated AgRP-GR KO mice were lower than Cort-treated control mice, but this effect was not as great as the effect of genotype.

Despite this difference, increases in the expression of genes involved in gluconeogenesis in liver after 3 weeks on Cort were similar between control and AgRP-GR KO mice (Fig. 4f). There were also similar responses to Cort in the control and AgRP-GR KO mice for genes involved in insulin signalling in skeletal muscle and liver after 3 weeks on Cort (Fig. 4g,h).

## Discussion

Chronic GC treatment can lead to many metabolic abnormalities. The present study has demonstrated that GCs acting on AgRP neurons contribute to the Cort-induced hyperinsulinaemia and hepatic steatosis in female mice. When these chronic effects of GCs on hypothalamic AgRP neurons are prevented in AgRP-GR KO mice, there were changes in mRNA expression consistent with enhanced adipose tissue de novo lipogenesis, decreased transport of fatty acids into the liver and reduced hepati﻿c de novo﻿ lipogenesis. It seems likely that these changes contribute to the reductions in hepatic steatosis and hyperinsulinaemia in the AgRP-GR KO mice. Additionally, the knockdown of GR on AgRP neurons leads to delayed onset Cort-induced hyperphagia, but with time, hyperphagia prevailed and there was similar body weight gain and adiposity to that in Cort treated control mice.

We have previously developed a robust and reproducible model of GC excess in male mice^9–11^. Here we characterised both male and female littermates and demonstrate a similar change in Cort-induced metabolic effects in female control (GR^flox/flox^) mice. Female mice developed hyperphagia, with an increase in body weight, increased BAT weight, decreased expression of genes involved in thermogenesis, and increased glucose stimulated insulin release. It is notable that in Cort treated female mice there was much less glucose excursion and faster normalisation of glucose after an ip glucose bolus. We can only speculate upon the tissue responsible for the improved glucose effectiveness seen in this paradigm of fasting and Cort treatment, and further dynamic studies of carbon sources relevant to gluconeogenic substrates and lipid flux are required to fully understand this finding. In keeping with the present study, previous studies using high dose Cort pellets also demonstrated a myriad of metabolic effects with Cort-treatment in female mice, but hyperglycaemia was also absent as well as a lower glucose excursion in female Cort treated mice during a GTT^33^. A further study reported no metabolic effects of GCs in female CD1 mice^34^. This observation may be due to mouse strain differences and/or the administration of a lower dose of Cort (50 μg/ml).

One of the most robust effects observed with GC treatment is hyperphagia, which is associated with elevated *Agrp* mRNA expression^9,11,35,36^. This effect would suggest that Cort treatment acts via increasing AgRP to cause elevated food intake, yet when AgRP is deleted globally, there is only a mild reduction in the GC induced hyperphagia^9^. In the present study, where GCs are unable to act on the whole AgRP neuron (also containing NPY and GABA) there was delayed onset hyperphagia in the AgRP-GR KO mice and they did not display the large increase in *Agrp* mRNA expression with Cort treatment. The role of the AgRP neurons in hyperphagia is supported by many studies where activation of AgRP neurons^21,22^ and increases in AgRP^37^ or NPY^38^ peptide levels are associated with hyperphagia in rodents. Conversely, AgRP neuron ablation in adult mice leads to starvation^39,40^. The phenomenon of an acute increase in leptin and insulin after GC treatment is well recognised^9,41^. Although both are considered to be anorexigenic drivers, the overall net integrated output in animals is still to drive hyperphagia. The mechanism underlying this dominant drive to eat remains to be determined.

The loss of GR from AgRP neurons afforded some protection from the Cort induced increase in food intake, at least in the earlier part of the study. However, this was not sufficient to reduce the Cort induced body weight gain and increases in adipose tissue mass measured at the end of the study. Though not measured in the present study, this is potentially due to changes in energy expenditure, as studies by another group have shown that female AgRP-GR KO mice have increased energy expenditure albeit on a high fat diet^32^. However, we observed no difference in BAT weight or the mRNA expression of markers of thermogenesis between Cort-treated WT and AgRP-GR KO mice.

In the present study where GCs were not able to signal in AgRP neurons, we showed improved GC-induced GSIS and fasting hyperinsulinaemia suggesting increased insulin sensitivity compared to Cort treated control mice. This is in keeping with previous studies where activation of AgRP neurons^27^ or overexpression of β-arrestin in these neurons^42^ leads to insulin resistance. In contrast, ablation of the neuron^43^ or modulation of intracellular signalling in AgRP neurons^29,42,44,45^ improves insulin sensitivity. This may be due to the potential signalling from AgRP neurons to the pancreas^43^. Equally, in a previous study, the deletion of purinergic receptor 6 (P2Y6) from AgRP neurons caused changes in AgRP neuronal inputs to the liver^44^, suggesting that in our AgRP-GR KO mice, the improvement in insulin sensitivity could additionally be related to signalling to the liver, leading to the lower levels of hepatic steatosis.

In the present study we observed a decrease in Cort-induced hepatic steatosis. This reduction in steatosis could be mediated, at least in part, by reduced AgRP neuron activity signalling to peripheral tissues. The AgRP-GR KO mice do not develop the elevated plasma triglycerides or liver weight on Cort and they have a reduction in hepatic lipids compared to control mice. Although previous studies on AgRP neurons have not investigated the effects of GCs, activation of AgRP neurons can affect substrate utilisation^23^ and mediate changes in liver and adipose tissue. Plasma triglycerides are increased when AgRP neurons are ablated^43^, whereas they are decreased when the enzyme, Crat, is deleted from AgRP neurons in mice on high fat diet^29^. Furthermore, liver triglycerides and hepatic lipid levels are increased when AgRP neurons are manipulated or ablated^29,42,43^. Additionally, manipulation of AgRP neurons can alter the genes associated with de novo lipogenesis and lipolysis in liver^29,42,43^. Moreover, manipulation of AgRP neurons has effects on adipose tissue lipolysis^23,29,46^, that could increase the availability of fatty acids for uptake into the liver in the Cort-treated control mice and this would be reduced in the AgRP-GR KO mice. Taken together these mechanisms may explain how the peripheral effects on lipid balance in AgRP-GR KO mice could be mediated by direct effects of AgRP neurons.

In conclusion, the knockdown of GR on AgRP neurons causes attenuation of the GC-induced increases in plasma and liver triglycerides, and partially prevents GC-induced hyperinsulinaemia and insulin signalling abnormalities in female mice. It also delayed the Cort-induced hyperphagia. This highlights the need to build an understanding of the role of chronic GCs acting on AgRP neurons which, at least in part, leads to the GC-induced hyperinsulinaemia and hepatic steatosis.

## Methods and materials

### Generation of genetically modified mice

All mice were bred in house at the University of Manchester, Manchester, UK. To generate mice with the glucocorticoid receptor (GR; Nr3c1) deleted solely from AgRP neurons we crossed GR^flox/flox^ (B6.129P2-Nr3c1tm2Gcs/Ieg EMMA strain #02124) with AgRP^-IRES-Cre^ mice (Agrp^tm1(cre)^Lowl/J Jackson strain #012899) to obtain AgRP^-IRES-Cre/+^::GR^flox/flox^ mice (hereinafter AgRP-GR KO), where GR was deleted solely from AgRP neurons. To fluorescently mark the AgRP neurons with eYFP, the AgRP-GR KO mice were crossed with R26-stop-eYFP mice (B6.129X1-GT(ROSA)26Sor^tm1(EYFP)Cos^/J Jackson stain #006148).

To genotype the mice, DNA was extracted from ear snips and mice were genotyped using the following primers: GR Flox: forward: 5′-GGC ATG CAC ATT ACT GGC CTT CT-3′, reverse 4 (NULL band) 5′-GTG TAG CAG CCA GCT TAC AGG A-3′ and reverse 8 (WT/Flox) 5′-CCT TCT CAT TCC ATG TCA GCA TGT-3′, giving product sizes of WT—225 bp, Flox—275 bp and NULL—390 bp. AgRP-Cre: common forward primer (12638) 5′-GCT TCT TCA ATG CCT TTT CG-3′, internal positive control (IPC) reverse primer (12639) 5′-GTG TGT GGT TCC AGC ATG AC-3′, and mutant reverse primer (12640) was 5′-AGG AAC TGC TTC CTT CAC GA-3′, giving an IPC product size of 199 bp in all mice and a mutant product size of 280 bp in the mice positive for Cre. ROSA26 genotyping used separate reactions for wild-type and mutant assays. The wild-type primers were forward (21306) 5′-CTG GCT TCT GAG GAC CG-3′ and reverse (24500) 5′-CAG GAC AAC GCC CAC ACA-3′ giving a product size of 142 bp. The mutant assay forward primer (24951) was 5′-AGG GCG AGG AGC TGT TCA-3′ and the reverse primer (24952) was 5′-TGA AGT CGA TGC CCT TCA G-3′ giving a product size of 384 bp. Heterozygous (Het) mice had a positive band in both the wild-type and the mutant assay.

### Study design

A total of 240 AgRP-GR KO mice and their control (GR^flox/flox^) littermate mice were used in these studies, with no deaths during the studies. AgRP^-IRES-Cre^ mice have been previously shown not to have a phenotype and were therefore not included as a control strain^42^. All mice were maintained on a 12:12 light cycle (lights on at 7 a.m. and lights off at 7 p.m.) in specific pathogen-free cages with wood chip bedding and environmental enrichment in an ambient temperature of 23 ± 1 °C. Food and water were available ad libitum, except where food was removed for fasting.

Nine to eleven week old mice were singly housed and acclimatised for 1 week, prior to a 2 week baseline during which food and water intake were monitored twice weekly. Mice were randomly assigned at genotyping to treatment group, either corticosterone (Cort; Sigma-Aldrich, 27840-500MG or Caymen Chemicals, 16063-500 mg-CAY both > 98% purity at 75 μg/ml in 1% ethanol) or vehicle (1% ethanol) in drinking water as in previous studies^9–11^. Animals were randomly assigned to the cage rack and were randomly moved during the course of the study. This prevented bias due to rack position. Three cohorts of mice were used. Male and female mice were treated for 21 days (main study), or female mice were treated for 48 h to investigate acute food intake and a further sub-group were treated for use in the ipGTT after 10 days treatment. All cohorts had a group size of 10. This was based on previous studies in a different strain of transgenic mice and accounted for the additional variability that we have previously observed with transgenic strains. In this study we powered based on a detectable change in body weight of 3.5 g, with a SD of 2.75. Using a group size of 10 gives us a power of 81.2. In the 21 day study, mice were tail bled for corticosterone prior to euthanasia by rising CO_2_ followed by exsanguination. Tissues were weighed and then snap frozen on dry ice, placed in RNAlater (Sigma, R0901), or placed in 10% formalin as appropriate. During the in-life measurements, researchers were blinded to the genotype of the animal, but the treatment was visible. Any samples taken from the mice were assigned a study number and from this point all measurements were fully blinded.

All experiments were performed in accordance with the UK Home Office legislation [Animal (Scientific Procedures) Act 1986], were approved by the University of Manchester local ethics committee and comply with the ARRIVE guidelines.

### Biochemical measurements

Glucose and insulin were measured fresh in tail-prick blood samples using a glucometer (Accu-Chek, Roche) and an ELISA (Crystal Chem, 90080) respectively. For corticosterone, blood samples were taken within one minute of disturbing the cage by tail-prick sample. Samples were then centrifuged, and the plasma frozen at − 80 °C for later analysis by ELISA (corticosterone, Abnova, KA0468). Triglycerides were measured using a colourimetric assay (Sigma, F6428 and T2449). This was carried out using plasma samples taken by cardiac puncture at the end of the study or frozen liver samples which were homogenised in propan-2-ol with Triton-X.

### Identification of the GR Flox null band in tissues

Mice were culled by rising CO_2_ and various tissues were snap frozen on dry ice. Genomic DNA (gDNA) was extracted from these tissues, the NULL band was amplified and visualised. The presence of a band indicates recombination, and therefore deletion, in that tissue. The primers were forward 5′-GGC ATG CAC ATT ACT GGC CTT CT-3′ and reverse (NULL) 5′-GTG TAG CAG CCA GCT TAC AGG A-3′. GAPDH was used as a loading control with forward 5′-AAC GAC CCC TTC ATT GA-3′ and reverse 5′-TCC ACG ACA TAC TCA GCA C-3′ primers. Samples were run on a 2% agarose gel and the GR NULL PCR gave a band of 390 bp and GAPDH 190 bp.

### Dual immunofluorescence for GR and AgRP (eYFP)

Female AgRP^CRE/+^::Rosa26^+/?^::GR^−/−^ (AgRP-GR KO x Rosa eYFP) mice and their AgRP^CRE/+^::Rosa26^+/?^::GR^+/+^ (GR WT Rosa eYFP) littermates were culled by rising CO_2._ Whole brains were dissected and placed into 4% paraformaldehyde for 24 h followed by 30% sucrose for 24–48 h as appropriate and rapidly frozen on dry ice. Brains were stored at −﻿ 80 °C until sectioning on a freezing microtome, where 25 μm sections were taken. Free-floating sections were washed in PBS-T before being permeabilised in 0.25% Triton-X. Tissues were incubated in 1% SDS then blocked in 5% goat serum with 0.5% BSA and 0.25% Triton-X for 1 h at room temperature. Sections were co-incubated with 1:5000 anti-GFP (Abcam, ab13970) and 1:300 Anti-GR (Proteintech, 24050-I-AP) primary antibodies for 3 h at room temperature followed by overnight at 4 °C. After PBS washes, sections were incubated with 1:400 goat anti-rabbit 594 (Life Technologies, A11037) and 1:400 goat anti-chicken 488 (Life Technologies, A11039) secondary antibodies in 5% goat serum, 0.5% BSA and 0.25% Triton-X for 2 h and 15 min at room temperature. Sections were washed in PBS followed by water, added to slides and dried before being mounted in Prolong Gold AntiFade mountant containing DAPI (Thermo Fisher, 11569306) and coverslipped.

Images were collected on a Zeiss Axioimager.D2 upright microscope using a 10×/EC Plan-neofluor objective and captured using a Coolsnap HQ2 camera (Photometrics) through Micromanager software v1.4.23. Images were then processed using ImageJ software (www.imagej.net). Four mice per genotype were stained and three to five sections per brain examined, leading to 18 sections per genotype being quantified for co-localisation of GR and eYFP.

### Real-time quantitative PCR

RT-qPCR was carried out as previously described in Sefton et al*.* 2019^9^. Briefly, RNA was extracted using an RNeasy Mini Kit (Qiagen, 74104) with on-column genomic DNA digestion, with an additional phenol extraction for adipose tissue. RNA was quantified using a Nanodrop-2000 spectrophotometer and was reverse transcribed using the High Capacity cDNA RT kit (Applied Biosystems, 4368814). Transcript levels were determined using a Prism 7900HT (Applied Biosystems) with either Universal Master Mix II (TaqMan; Applied Biosystems, 4440043) or Go Taq qPCR Master Mix (SYBR; Promega, A6010). TaqMan catalogue numbers and primer sequences are listed in Supplemental Tables 1 and 2. Samples were quantified using a standard curve with HPRT or TBP for TaqMan or SYBR assays, respectively, as reference genes, and the WT vehicle group as calibrator.

### Immunohistochemistry

Livers were fixed in formalin for 24 h, cryoprotected in 30% sucrose for 24 h and frozen in OCT using supercooled 2-methylbutane, and stored at − 80 °C. Livers were sectioned at 12 μm and were taken at 3 depths through the tissue. Slides were frozen at − 80 °C prior to staining. Hemotoxylin & eosin staining was performed as before^9^. For Oil Red O staining, slides were incubated with Oil Red O (Sigma, CI 26125) without a counterstain, prior to mounting and cover slipping.

All images were visualised using a 20×/0.80 Plan Apo objective using a 3D-Histech Panoramic-250 Flash II slide scanner (3D Histech, Hungary). Snapshots of the slide scans were taken using CaseViewer software (3D Histech).

Oil Red O staining was quantified using ImageJ software. Ten fields of view per section (30 fields of view per animal) were analysed to give one value per mouse.

### Intraperitoneal glucose tolerance test

Mice were given Cort in drinking water for 10 days before undergoing an intraperitoneal glucose tolerance test (ipGTT). Food was removed and mice were placed in clean cages at 8am for a 5 h fast. Before the ipGTT mice were weighed and had a T = 0 blood sample taken by tail-prick microsampling for glucose (blood droplet, Accu-Chek) and insulin (5 μl blood, ELISA, Crystal Chem, 90,080). Mice were given an ip injection of 20% glucose (2 g/kg fasted body weight). Further samples were taken at T = 15 min and T = 30 min post glucose for glucose and insulin and T = 60 min and T = 120 min for glucose only.

### Statistical analysis

The data were analysed using Prism 8.0 (GraphPad) and are presented as mean ± S.E.M. For normally distributed data, Student’s *t-*test was performed between two groups and a two-way ANOVA between multiple groups. For non-normally distributed data (including real-time quantitative PCR data), the groups were compared by Mann–Whitney *U* test. *P* values of 0.05 were considered to indicate statistical significance. Samples that were 2 SD ± mean were removed as statistical outliers which was decided prior to the commencement of the study.

## Supplementary information

Supplementary Information.

## Data Availability

The datasets generated during and/or analysed during the current study are available from the corresponding author on reasonable request.
