# Supplementary material for: Chronic glucocorticoid treatment induces hepatic lipid accumulation and hyperinsulinaemia in part through actions on AgRP neurons
Source: Sci Rep. 2021 Jul 2;11:13776. doi: 10.1038/s41598-021-93378-3 (PMC8253818; doi:10.1038/s41598-021-93378-3)

## Slide 1
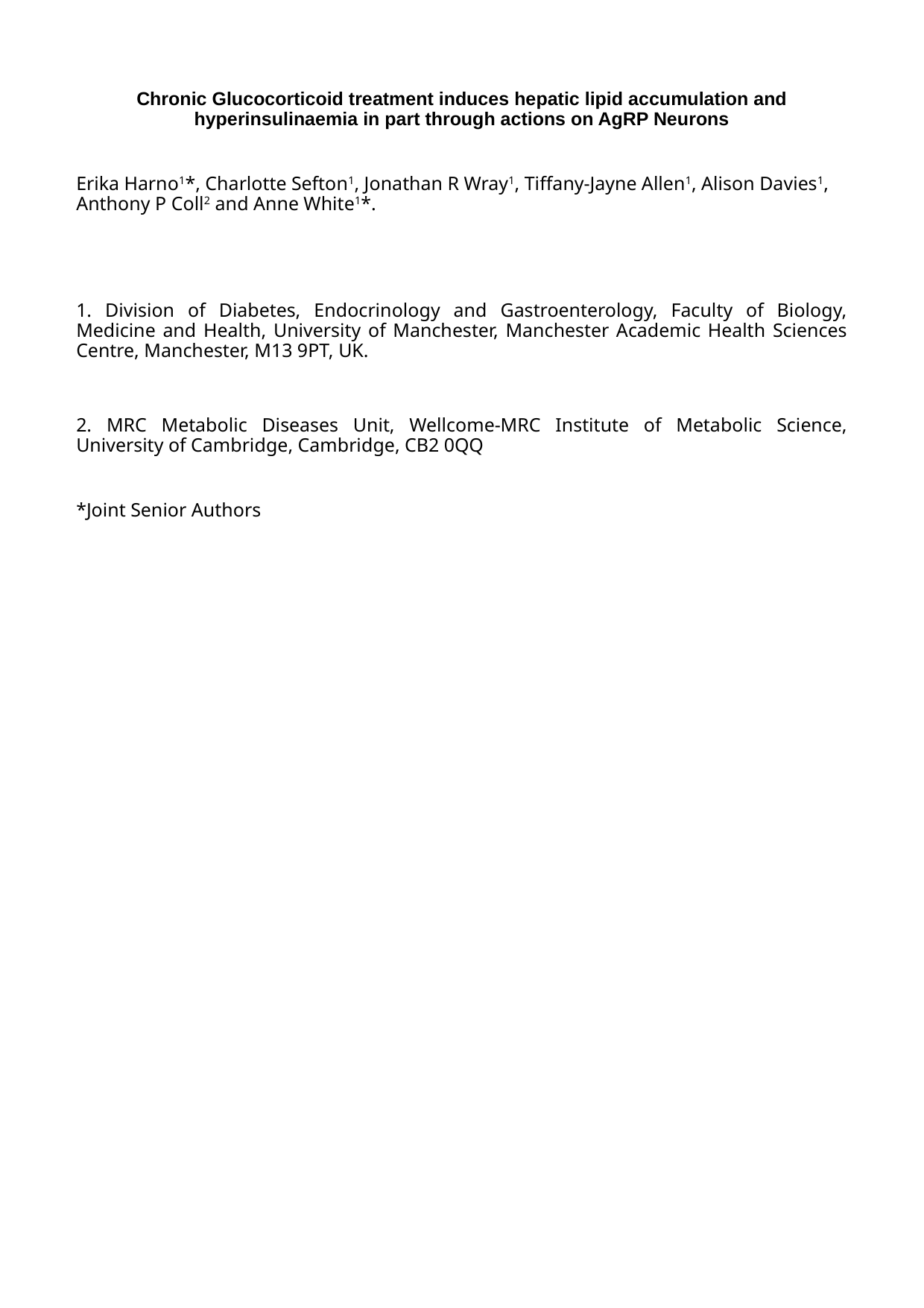

Chronic Glucocorticoid treatment induces hepatic lipid accumulation and hyperinsulinaemia in part through actions on AgRP Neurons
Erika Harno1*, Charlotte Sefton1, Jonathan R Wray1, Tiffany-Jayne Allen1, Alison Davies1, Anthony P Coll2 and Anne White1*.
1. Division of Diabetes, Endocrinology and Gastroenterology, Faculty of Biology, Medicine and Health, University of Manchester, Manchester Academic Health Sciences Centre, Manchester, M13 9PT, UK.
2. MRC Metabolic Diseases Unit, Wellcome-MRC Institute of Metabolic Science, University of Cambridge, Cambridge, CB2 0QQ
*Joint Senior Authors

## Slide 2
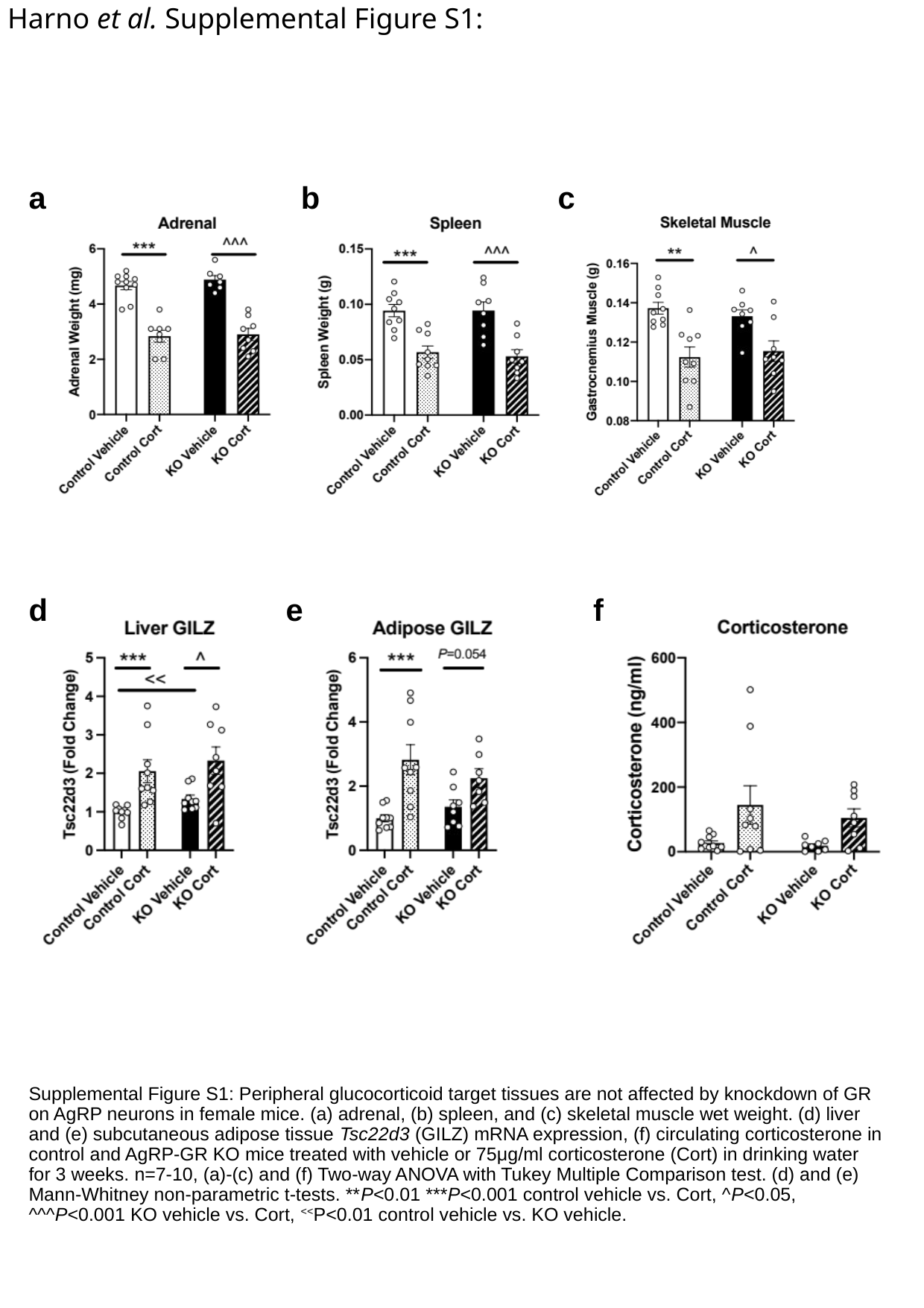

Harno et al. Supplemental Figure S1:
c
a
b
e
f
d
Supplemental Figure S1: Peripheral glucocorticoid target tissues are not affected by knockdown of GR on AgRP neurons in female mice. (a) adrenal, (b) spleen, and (c) skeletal muscle wet weight. (d) liver and (e) subcutaneous adipose tissue Tsc22d3 (GILZ) mRNA expression, (f) circulating corticosterone in control and AgRP-GR KO mice treated with vehicle or 75μg/ml corticosterone (Cort) in drinking water for 3 weeks. n=7-10, (a)-(c) and (f) Two-way ANOVA with Tukey Multiple Comparison test. (d) and (e) Mann-Whitney non-parametric t-tests. **P<0.01 ***P<0.001 control vehicle vs. Cort, ^P<0.05, ^^^P<0.001 KO vehicle vs. Cort, <<P<0.01 control vehicle vs. KO vehicle.

## Slide 3
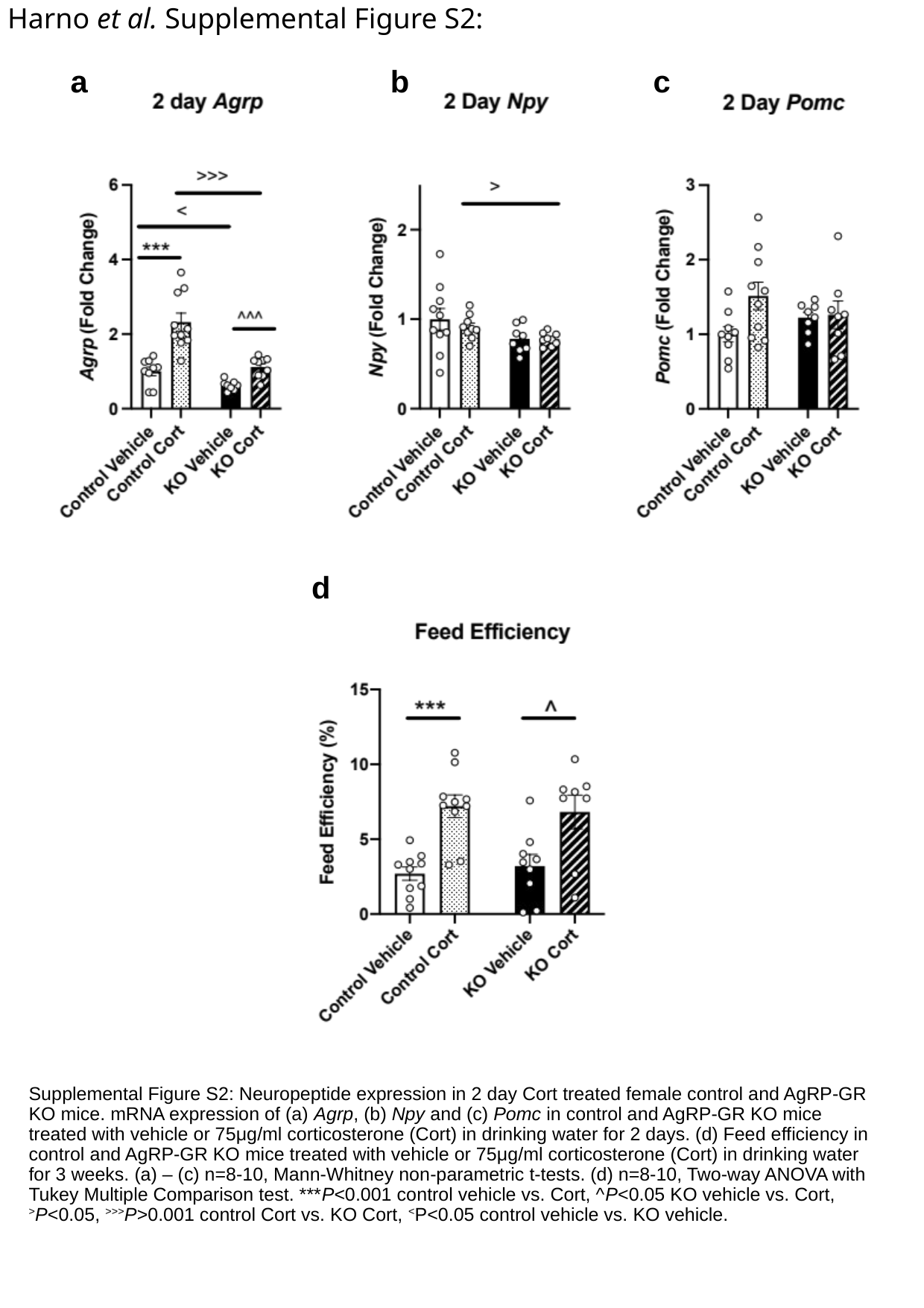

Harno et al. Supplemental Figure S2:
a
c
b
d
Supplemental Figure S2: Neuropeptide expression in 2 day Cort treated female control and AgRP-GR KO mice. mRNA expression of (a) Agrp, (b) Npy and (c) Pomc in control and AgRP-GR KO mice treated with vehicle or 75μg/ml corticosterone (Cort) in drinking water for 2 days. (d) Feed efficiency in control and AgRP-GR KO mice treated with vehicle or 75μg/ml corticosterone (Cort) in drinking water for 3 weeks. (a) – (c) n=8-10, Mann-Whitney non-parametric t-tests. (d) n=8-10, Two-way ANOVA with Tukey Multiple Comparison test. ***P<0.001 control vehicle vs. Cort, ^P<0.05 KO vehicle vs. Cort, >P<0.05, >>>P>0.001 control Cort vs. KO Cort, <P<0.05 control vehicle vs. KO vehicle.

## Slide 4
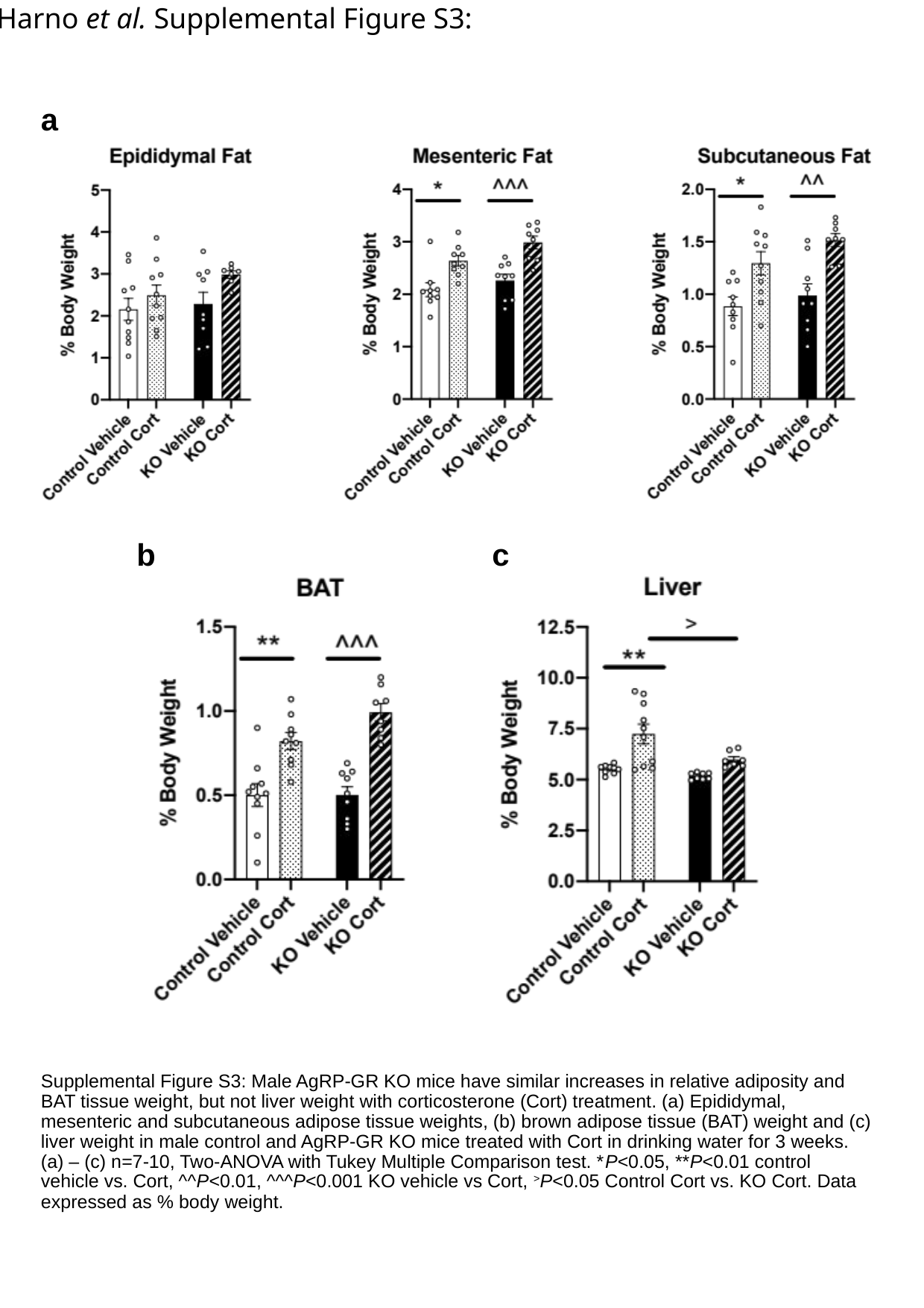

Harno et al. Supplemental Figure S3:
a
b
c
Supplemental Figure S3: Male AgRP-GR KO mice have similar increases in relative adiposity and BAT tissue weight, but not liver weight with corticosterone (Cort) treatment. (a) Epididymal, mesenteric and subcutaneous adipose tissue weights, (b) brown adipose tissue (BAT) weight and (c) liver weight in male control and AgRP-GR KO mice treated with Cort in drinking water for 3 weeks. (a) – (c) n=7-10, Two-ANOVA with Tukey Multiple Comparison test. *P<0.05, **P<0.01 control vehicle vs. Cort, ^^P<0.01, ^^^P<0.001 KO vehicle vs Cort, >P<0.05 Control Cort vs. KO Cort. Data expressed as % body weight.

## Slide 5
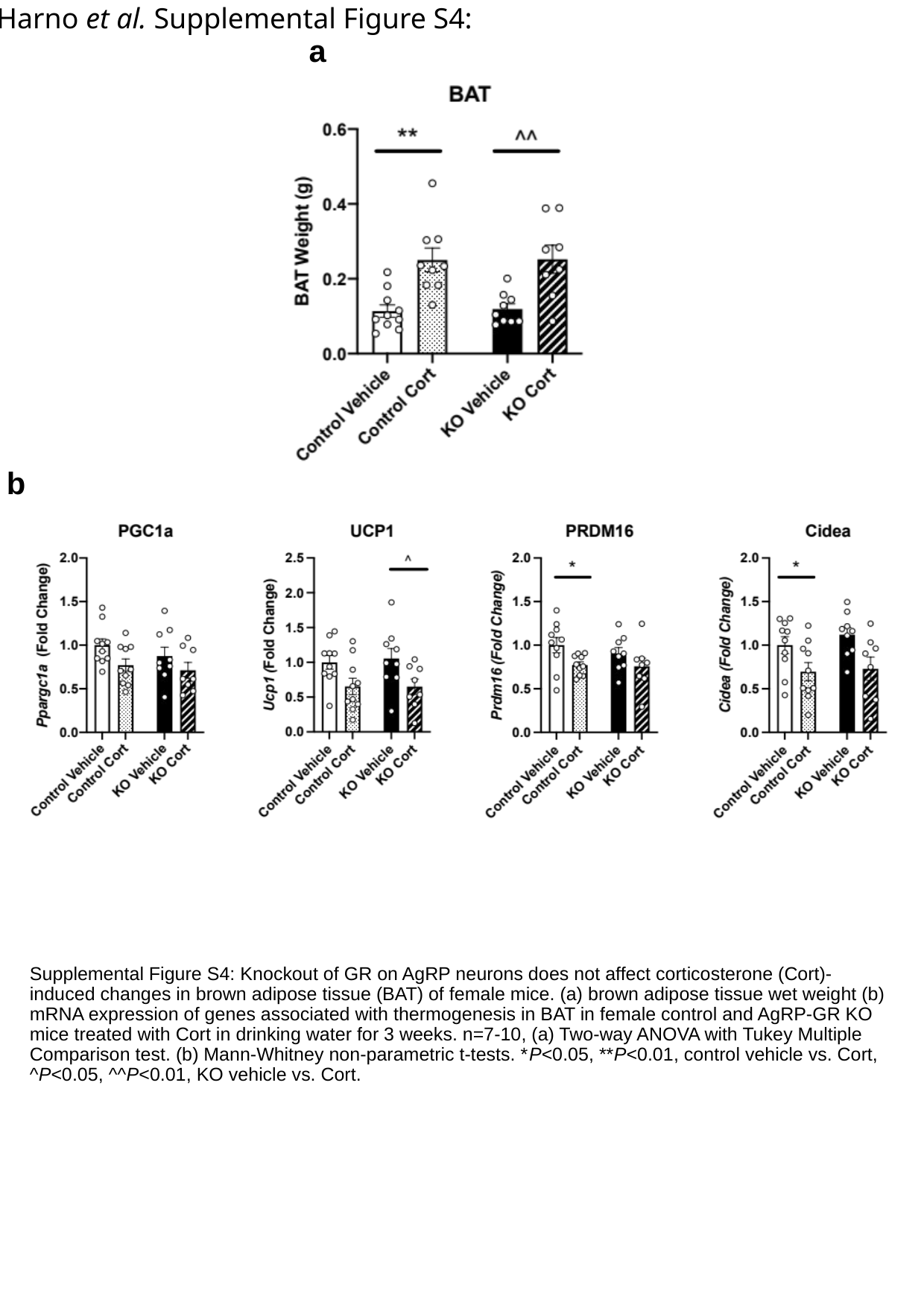

Harno et al. Supplemental Figure S4:
a
b
Supplemental Figure S4: Knockout of GR on AgRP neurons does not affect corticosterone (Cort)-induced changes in brown adipose tissue (BAT) of female mice. (a) brown adipose tissue wet weight (b) mRNA expression of genes associated with thermogenesis in BAT in female control and AgRP-GR KO mice treated with Cort in drinking water for 3 weeks. n=7-10, (a) Two-way ANOVA with Tukey Multiple Comparison test. (b) Mann-Whitney non-parametric t-tests. *P<0.05, **P<0.01, control vehicle vs. Cort, ^P<0.05, ^^P<0.01, KO vehicle vs. Cort.

## Slide 6
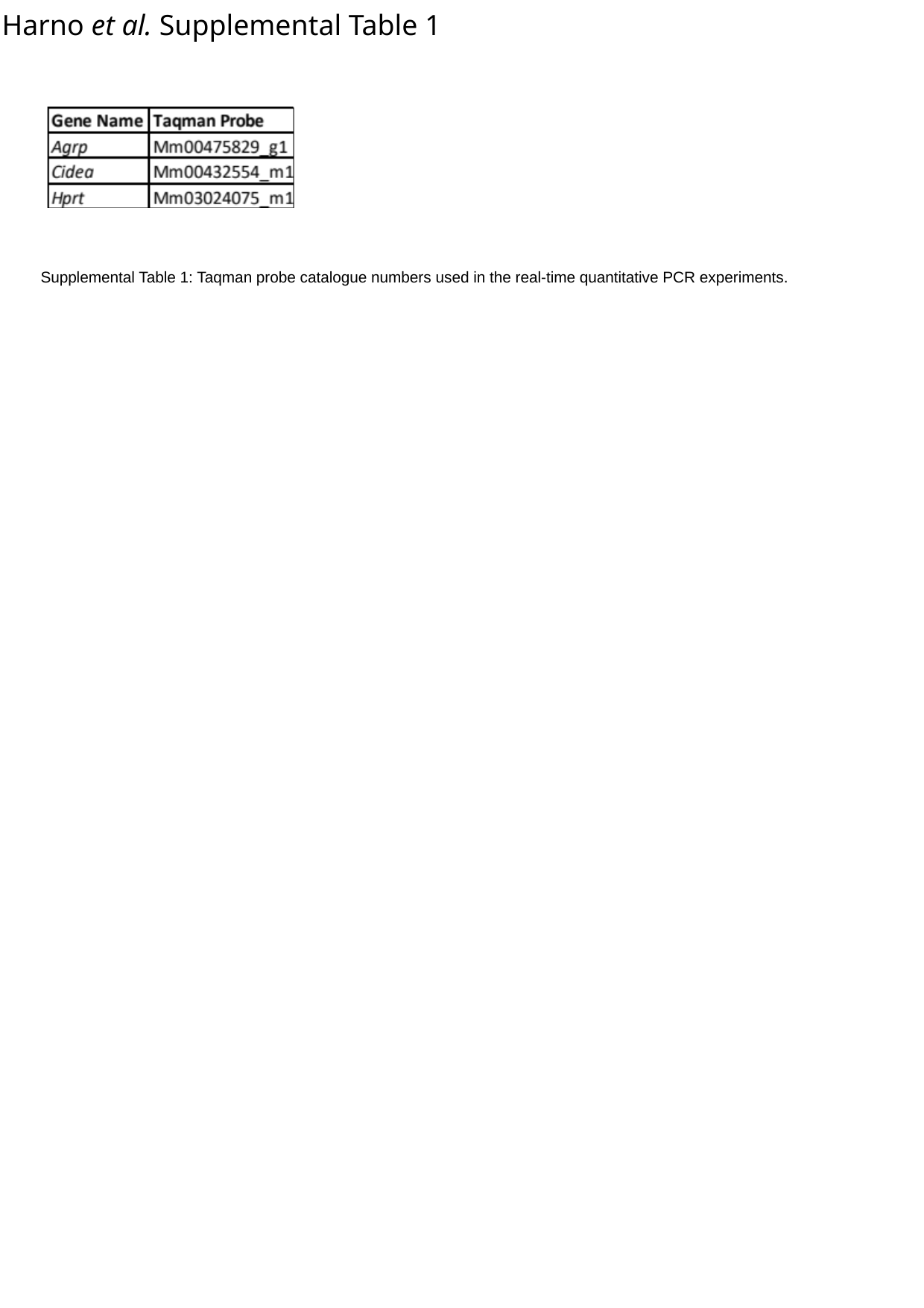

Harno et al. Supplemental Table 1
Supplemental Table 1: Taqman probe catalogue numbers used in the real-time quantitative PCR experiments.

## Slide 7
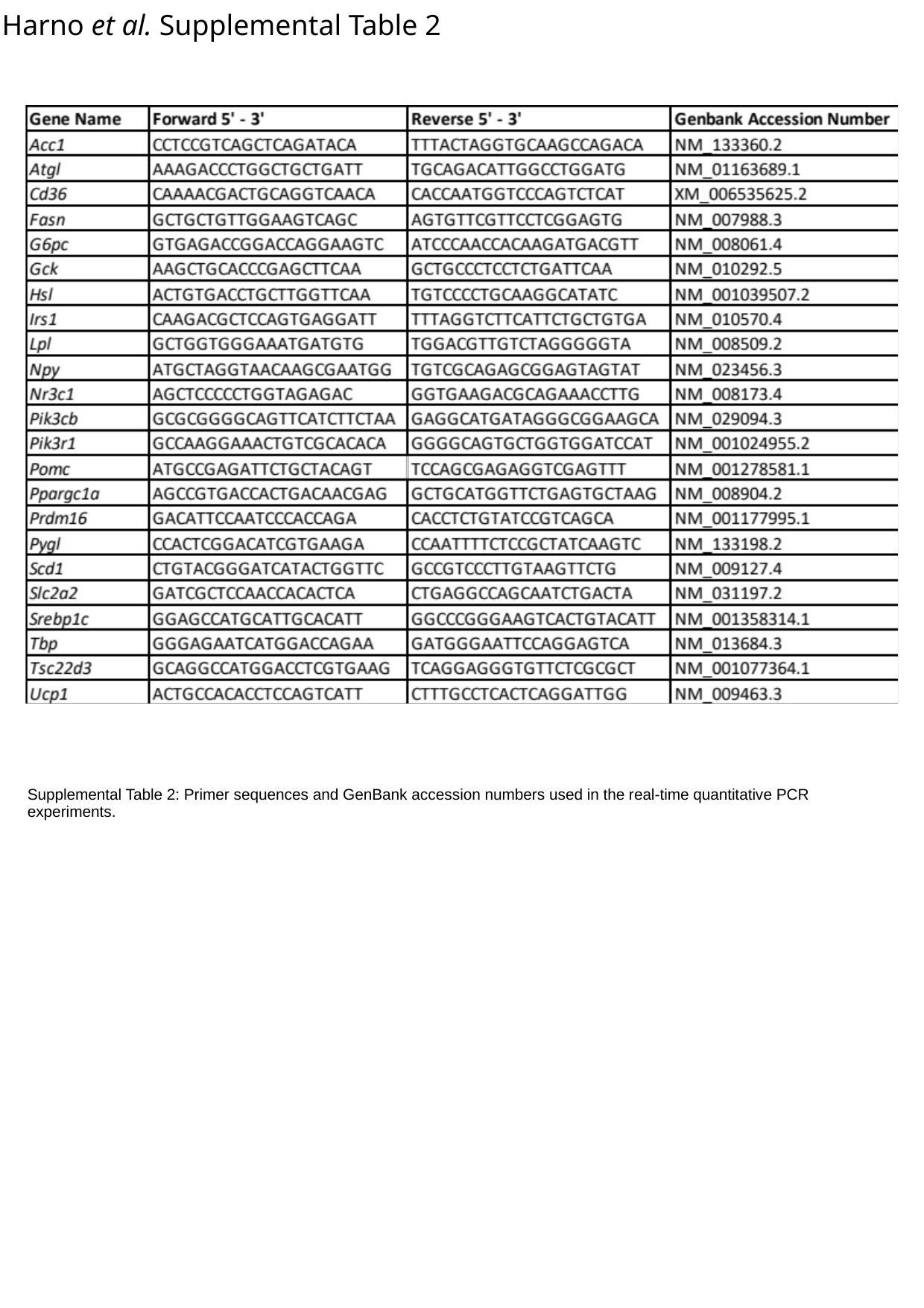

Harno et al. Supplemental Table 2
Supplemental Table 2: Primer sequences and GenBank accession numbers used in the real-time quantitative PCR experiments.

## Slide 8
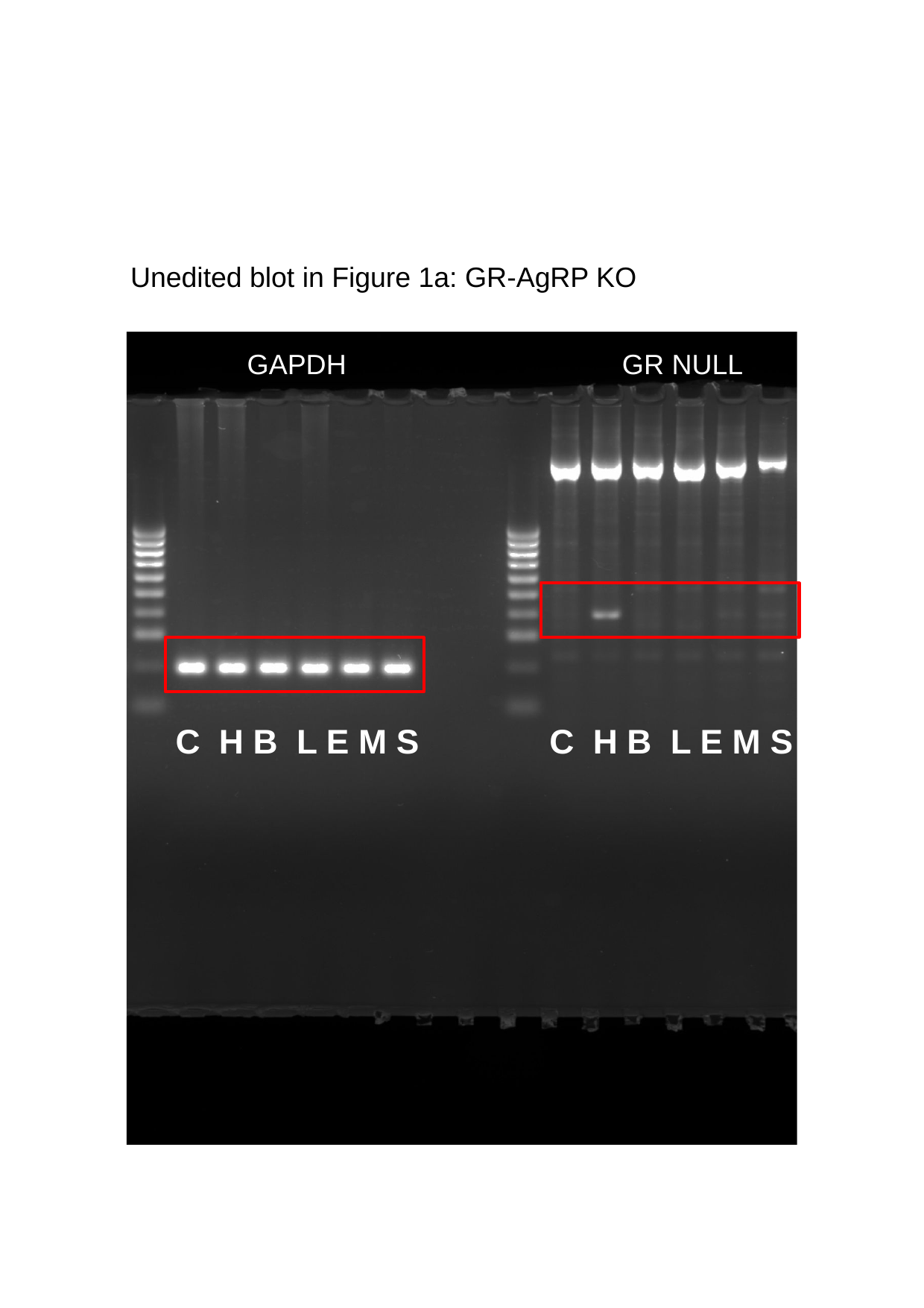

Unedited blot in Figure 1a: GR-AgRP KO
GAPDH
GR NULL
 C H B L E M S
 C H B L E M S

## Slide 9
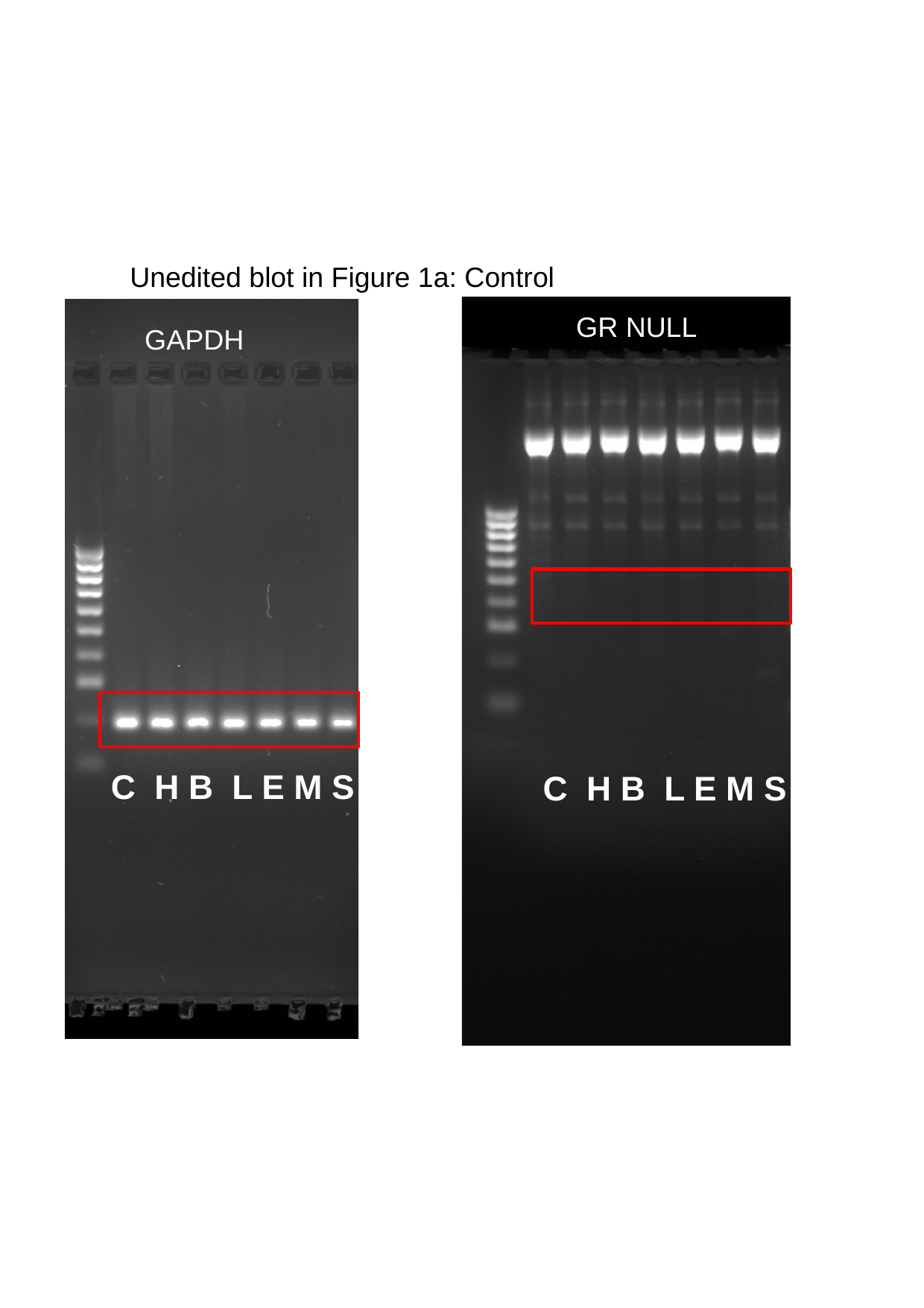

Unedited blot in Figure 1a: Control
GR NULL
GAPDH
 C H B L E M S
 C H B L E M S

## Slide 10
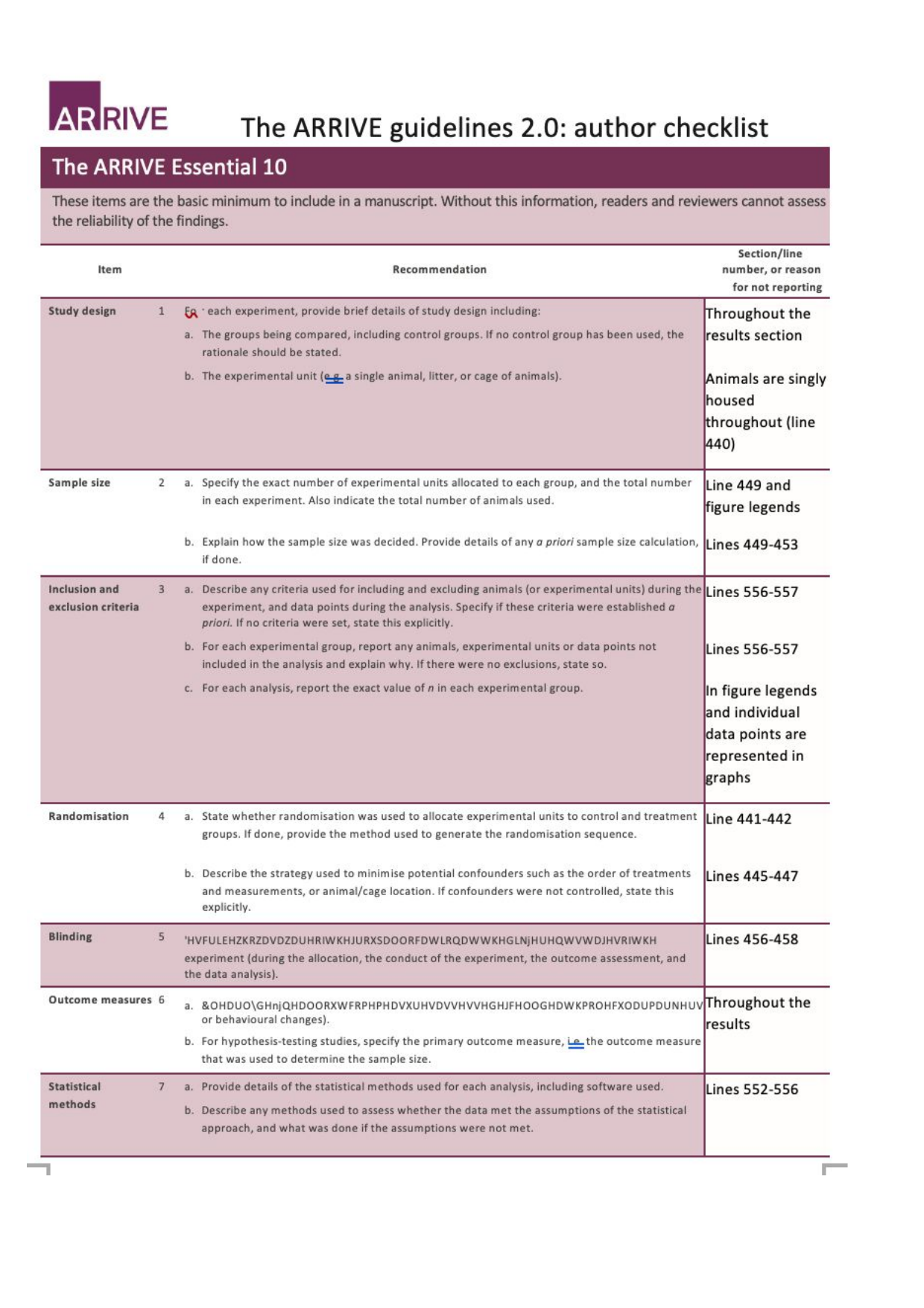

## Slide 11
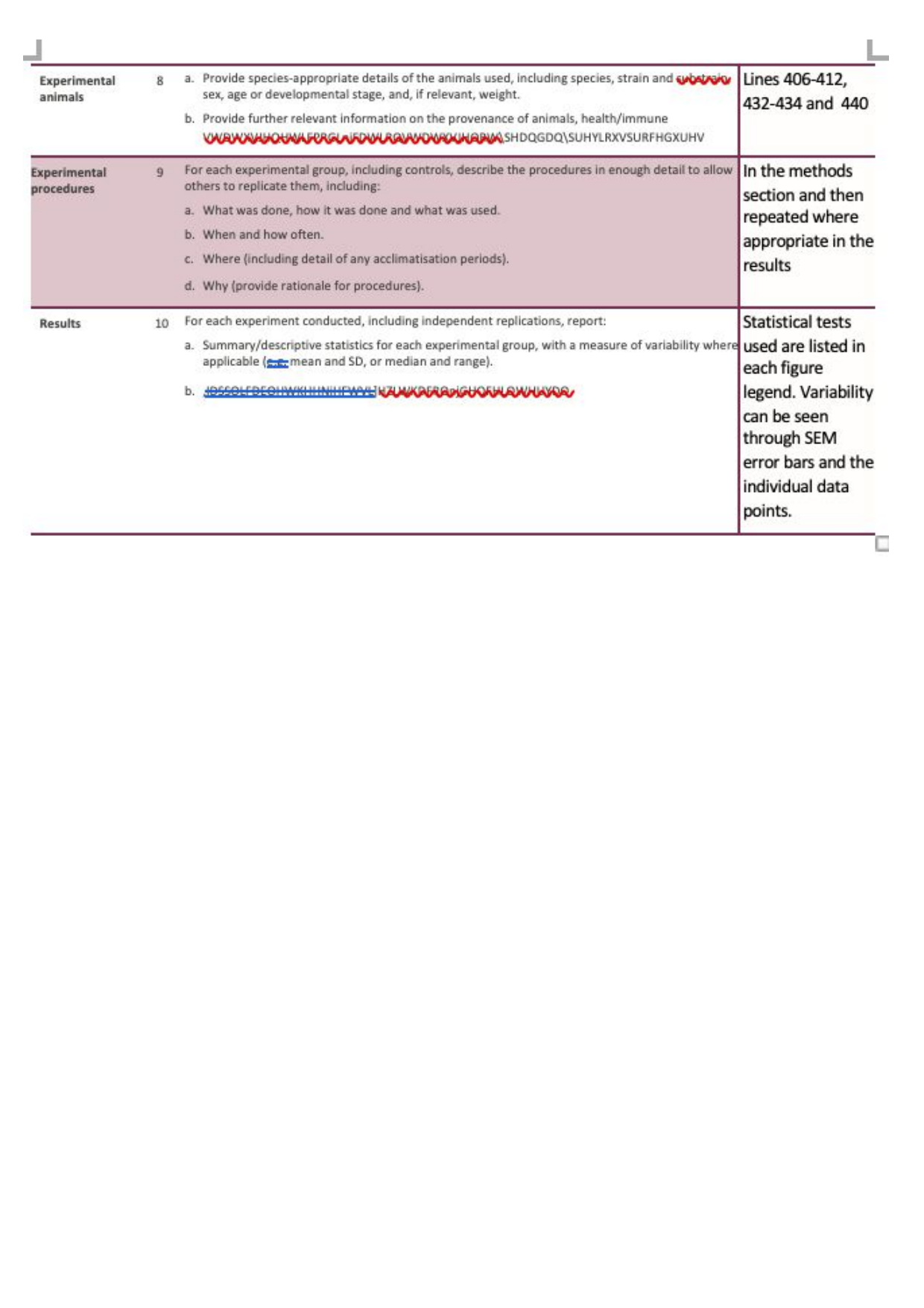

Supplement: Supplementary file 1 — Supplementary Information. [file 41598_2021_93378_MOESM1_ESM.pptx]
